# Endothelin B Receptor Immunodynamics in Pulmonary Arterial Hypertension

**DOI:** 10.3389/fimmu.2022.895501

**Published:** 2022-06-09

**Authors:** Christoph Tabeling, Carla R. González Calera, Jasmin Lienau, Jakob Höppner, Thomas Tschernig, Olivia Kershaw, Birgitt Gutbier, Jan Naujoks, Julia Herbert, Bastian Opitz, Achim D. Gruber, Berthold Hocher, Norbert Suttorp, Harald Heidecke, Gerd-R. Burmester, Gabriela Riemekasten, Elise Siegert, Wolfgang M. Kuebler, Martin Witzenrath

**Affiliations:** ^1^ Division of Pulmonary Inflammation, Charité - Universitätsmedizin Berlin, Corporate Member of Freie Universität Berlin and Humboldt-Universität zu Berlin, Berlin, Germany; ^2^ Department of Infectious Diseases and Respiratory Medicine, Charité - Universitätsmedizin Berlin, Corporate Member of Freie Universität Berlin and Humboldt-Universität zu Berlin, Berlin, Germany; ^3^ Berlin Institute of Health at Charité - Universitätsmedizin Berlin, Berlin, Germany; ^4^ Department of Rheumatology and Clinical Immunology, Charité - Universitätsmedizin Berlin, Corporate Member of Freie Universität Berlin and Humboldt-Universität zu Berlin, Berlin, Germany; ^5^ Institute of Anatomy and Cell Biology, University of Saarland, Homburg, Germany; ^6^ Department of Veterinary Pathology, Freie Universität Berlin, Berlin, Germany; ^7^ Fifth Department of Medicine (Nephrology/Endocrinology/Rheumatology), University of Heidelberg, University Medical Centre Mannheim, Heidelberg, Germany; ^8^ Key Laboratory of Study and Discovery of Small Targeted Molecules of Hunan Province, School of Medicine, Hunan Normal University, Changsha, China; ^9^ Reproductive and Genetic Hospital of CITIC-Xiangya, Changsha, China; ^10^ German Center for Lung Research (DZL), Partner Site Charité, Berlin, Germany; ^11^ CellTrend GmbH, Luckenwalde, Germany; ^12^ Department of Rheumatology, University of Lübeck, Lübeck, Germany; ^13^ Institute of Physiology, Charité - Universitätsmedizin Berlin, Corporate Member of Freie Universität Berlin and Humboldt-Universität zu Berlin, Berlin, Germany; ^14^ German Center for Cardiovascular Research (DZHK), Partner Site, Berlin, Germany; ^15^ St. Michael’s Hospital, Keenan Research Centre for Biomedical Science, Toronto, ON, Canada; ^16^ Departments of Physiology and Surgery, University of Toronto, Toronto, ON, Canada

**Keywords:** endothelin B receptor, autoantibody, Th2 inflammation, pulmonary vascular hyperresponsiveness, pulmonary arterial hypertension, systemic sclerosis

## Abstract

**Introduction:**

Inflammation is a major pathological feature of pulmonary arterial hypertension (PAH), particularly in the context of inflammatory conditions such as systemic sclerosis (SSc). The endothelin system and anti-endothelin A receptor (ET_A_) autoantibodies have been implicated in the pathogenesis of PAH, and endothelin receptor antagonists are routinely used treatments for PAH. However, immunological functions of the endothelin B receptor (ET_B_) remain obscure.

**Methods:**

Serum levels of anti-ET_B_ receptor autoantibodies were quantified in healthy donors and SSc patients with or without PAH. Age-dependent effects of overexpression of prepro-endothelin-1 or ET_B_ deficiency on pulmonary inflammation and the cardiovascular system were studied in mice. Rescued ET_B_-deficient mice (ET_B_^-/-^) were used to prevent congenital Hirschsprung disease. The effects of pulmonary T-helper type 2 (Th2) inflammation on PAH-associated pathologies were analyzed in ET_B_^-/-^ mice. Pulmonary vascular hemodynamics were investigated in isolated perfused mouse lungs. Hearts were assessed for right ventricular hypertrophy. Pulmonary inflammation and collagen deposition were assessed *via* lung microscopy and bronchoalveolar lavage fluid analyses.

**Results:**

Anti-ET_B_ autoantibody levels were elevated in patients with PAH secondary to SSc. Both overexpression of prepro-endothelin-1 and rescued ET_B_ deficiency led to pulmonary hypertension, pulmonary vascular hyperresponsiveness, and right ventricular hypertrophy with accompanying lymphocytic alveolitis. Marked perivascular lymphocytic infiltrates were exclusively found in ET_B_^-/-^ mice. Following induction of pulmonary Th2 inflammation, PAH-associated pathologies and perivascular collagen deposition were aggravated in ET_B_^-/-^ mice.

**Conclusion:**

This study provides evidence for an anti-inflammatory role of ET_B_. ET_B_ seems to have protective effects on Th2-evoked pathologies of the cardiovascular system. Anti-ET_B_ autoantibodies may modulate ET_B_-mediated immune homeostasis.

## Introduction

Despite modern therapy, pulmonary arterial hypertension (PAH) remains a fatal condition. PAH is characterized by construction and remodelling of pulmonary arteries leading to increased pulmonary vascular resistance and right heart failure (1).

An increasing body of evidence points to inflammation as a central pathogenic factor in idiopathic PAH (iPAH) as well as PAH secondary to other conditions (2–8). Perivascular inflammation and lymphoid tissue are found in lungs of PAH patients, and concordantly in murine models of pulmonary hypertension (2, 9–12). Elevated numbers of mast cells and T-helper type 2 (Th2) lymphocytes as well as increased expression of Th2 cytokines were repeatedly found in patients with pulmonary hypertension (2, 8, 13–16). Analogously, preclinical studies indicate an important underlying role of Th2-mediated immune signaling in the induction of morphological and functional changes found in PAH (2, 16–23).

The concept that the endothelium-derived peptide endothelin-1 (ET-1) serves as a major driver of PAH pathobiology is broadly accepted (1, 24–26). Prepro-endothelin-1 is the precursor of big-ET-1, which is converted to mature, bioactive ET-1 (24, 26, 27). Patients with pulmonary hypertension show elevated pulmonary vascular expression of ET-1 as well as increased levels of circulating ET-1 (25, 28).

ET-1 is a potent vasoconstrictor in the pulmonary circulation through activation of the G protein-coupled receptors endothelin A receptor (ET_A_) and endothelin B receptor (ET_B_) expressed on pulmonary arterial smooth muscle cells (PASMCs) (26, 27). The vasoconstrictive response induced by ET-1 involves thromboxane A_2_ (TXA_2_) release and consecutive TXA_2_ receptor activation (29, 30). Downstream signalling of ET-1-evoked vasoconstriction critically depends on protein kinase C isozyme alpha (PKCα) (31). Contrariwise, activation of ET_B_ located on endothelial cells induces vasodilation *via* release of nitric oxide (NO) and prostaglandins (26, 27), partially dampening the vasoconstrictive effects of ET-1.

Besides its vasomotor actions, ET-1 promotes immune cell trafficking (32), such as *via* release of tumor necrosis factor alpha (TNF-α), interleukin (IL)-1β, and IL-6 from monocytes and macrophages (33), or *via* ET_A_-dependent release of IL-6 from vascular smooth muscle cells (34).

Endothelin receptor antagonists (ERAs) are routinely used for the treatment of PAH. However, whether ET_A_-selective targeting or dual inhibition of ET_A_/ET_B_ is superior to the other is controversially discussed.

In systemic sclerosis (SSc), PAH is a common vascular complication, and an important driver of mortality (24). The prognosis of PAH secondary to SSc (SSc-PAH) has been critically linked to the presence of anti-ET_A_ autoantibodies (AAb) (35). Autoimmunity is believed to play a significant role in PAH pathobiology (2, 5, 6, 35–40), and blood plasmablast levels were shown to be elevated in PAH patients (36). However, the potential involvement of anti-ET_B_ AAb in PAH is currently unknown and the immunomodulatory role of ET_B_ in the context of pulmonary hypertension remains largely elusive.

In this study, anti-ET_B_ AAb levels were assessed in PAH patients for the first time. To better characterize the immunomodulatory functions of ET_B_, we additionally studied the effects of rescued ET_B_ deficiency in mice on pulmonary vascular disease, independent of and dependent on pulmonary Th2 inflammation.

Circulating ET-1 is cleared from the blood *via* ET-1/ET_B_ complex internalization (27, 41–43) and ET_B_ deficiency results in increased ET-1 plasma levels (44–46). Thus, effects on PAH-associated cardiovascular pathologies were studied in parallel in mice overexpressing prepro-ET-1 (_pre_ET^tg^) to allow differentiation between ET-1- and ET_B_-mediated effects.

We hypothesized that ET_B_ plays an anti-inflammatory role, alleviating Th2-evoked pathologies of the cardiovascular system.

## Materials and Methods

### Patients and Clinical Manifestations

Serum samples from 177 SSc patients referred to the Department of Rheumatology and Clinical Immunology at Charité - Universitätsmedizin Berlin were collected. Patients with SSc met the American College of Rheumatology/European League Against Rheumatism 2013 classification (47). SSc patients were classified as having either limited cutaneous SSc or diffuse cutaneous SSc or SSc sine scleroderma, according to the LeRoy criteria, depending on the distribution and history of skin sclerosis at the study visit. The first non-Raynaud symptom was considered as disease onset.

Under clinical routine conditions, patients were screened for PAH at least in 1-year intervals by assessment of World Health Organization functional class, echocardiography, lung function including single-breath diffusion capacity for carbon monoxide (DLCO_SB_), and, during the last few years, also by the detection of N-terminal pro-brain natriuretic peptide (NT-proBNP) levels. In all SSc patients in which PAH was suspected, diagnosis was confirmed by right heart catheterization. Interstitial lung disease was identified on the basis of a high-resolution computed tomographic scan, as confirmed by an expert radiologist. Additional serum samples were obtained from 10 iPAH patients, confirmed by right heart catheterization. Control serum samples were obtained from 26 healthy subjects.

The epidemiologic data of patients and healthy donors are shown in **Supplementary Table 1**. The study protocol was approved by the ethics committee (Charité - Universitätsmedizin Berlin; EA1/179/17). A written informed consent was obtained from each patient. The study was conducted in accordance with the principles of the Declaration of Helsinki.

### Detection of Anti-ET_B_ AAb

Prior to analysis, serum samples were stored at −80°C. Serum ET_B_ antibody levels were measured in duplicate by ELISA (CellTrend GmbH, Luckenwalde, Germany) in a blinded manner as described (35, 48). In brief, microtiter plates were coated with extracts from Chinese hamster ovary cells overexpressing human ET_B_. Calcium chloride (1 mmol/L) was administered to each buffer for maintenance of conformational epitopes. Diluted serum samples were incubated (1:100, 4°C, 2 h). For detection, plates were washed and incubated for 1 h with horseradish peroxidase-labeled goat anti-human immunoglobulin G (IgG; 1:20,000; Jackson, West Grove, Pennsylvania, USA), followed by enzymatic substrate reaction. Optical densities were converted into concentrations (U/ml) by comparison to a standard curve. Concentrations below the limit of detection (LOD) were depicted as LOD/√2.

### Mice

All animal procedures were approved by institutional authorities of the Charité - Universitätsmedizin Berlin and the Local State Office of Health and Social Affairs Berlin (LAGeSo; Berlin, Germany). Transgenic mice overexpressing human prepro-ET-1 (_pre_ET^tg^) on a mixed NMRI/C57BL/6 background rescued endothelin B receptor-deficient mice (ET_B_^-/-^) on a mixed C57BL/6/129 background and the respective corresponding wild-type mice were housed under specific pathogen-free conditions. The generation of _pre_ET^tg^ mice (49) and rescued ET_B_-deficient mice (50) has been described elsewhere. Rescued ET_B_-deficient mice hold a dopamine-β-hydroxylase ET_B_ transgene to prevent fatal congenital Hirschsprung disease (50).

### Isolated Perfused Mouse Lung

Here, we used the isolated perfused mouse lung preparation to evaluate pulmonary hemodynamics *ex vivo*. While this approach does not allow determining whether a specific model fulfills the clinical criteria of pulmonary hypertension *in vivo* (which is, however, obscured anyway by the fact that invasive hemodynamic measurements in mice are commonly restricted to recordings of right ventricular systolic pressure rather than mean Ppa), constant perfusion rates and defined left atrial pressures allow for a sensitive assessment of differences in pulmonary vascular resistance independent of right and left ventricular function.

Anesthetized mice were prepared, lungs were isolated, and pulmonary artery and left atrium were cannulated as described (51–53). Lungs were perfused constantly (1 mL/min, nonrecirculating, left atrial pressure 2.2 cmH_2_O) with 37°C sterile Krebs-Henseleit hydroxyethyl amylopectin buffer (Serag-Wiessner, Naila, Germany). Negative pressure ventilation was performed (*P*exp −4.5, *P*ins −9.0 cmH_2_O, 90 breaths/min). Pulmonary arterial pressure (Ppa) and dynamic lung compliance (C_dyn_) were measured and recorded *via* Pulmodyn software (17). ET-1, thromboxane analog U46619, or serotonin (all Merck KGaA, Darmstadt, Germany) was administered to the perfusion buffer for 10, 3, or 0.5 min, respectively (31). Doses were increased following intervals of 24 (ET-1), 12 (U46619), or 8 min (serotonin). Vasopressor responses were calculated (maximal difference in Ppa, Δ Ppa). To determine the role of ET_A_, ET_A_ inhibitor BQ-123 (8 μmol/L; Merck KGaA) or solvent (aqua dest.) was added to the perfusion buffer 10 min prior to ET-1 application. Lungs with signs of edema, atelectasis, or hemostasis were excluded from further analyses.

### Fulton Index

Hearts were excised. Right ventricle and left ventricle plus adjacent septum were microscopically dissected and weighed. Fulton index [quotient of right ventricle (RV) and left ventricle (LV) including septum (S)] was calculated.

### Pulmonary Th2 Inflammation

After systemic sensitization *via* i.p. injections of 20 μg of ovalbumin (OVA; grade V; Merck KGaA) dissolved in 100 µL of aluminum hydroxide suspension (1.3%; SERVA Electrophoresis GmbH, Heidelberg, Germany) and 10 µL of phosphate-buffered saline (PBS) on days 0 and 14, mice were repeatedly exposed to aerosolized ovalbumin (1%) in PBS on days 28–30 for 20 min/day (53, 54). On the respective days, sham-treated mice received i.p. injections of 100 µL of aluminum hydroxide suspension and 10 µL of PBS, followed by exposure to aerosolized PBS. Effects of pulmonary Th2 inflammation were analyzed on day 32.

### Bronchoalveolar Lavage

Bronchoalveolar lavage (BAL) of the right lung was performed twice with 650 µL of ice-cold PBS containing protease inhibitor (cOmplete™ mini; Merck KGaA) (55, 56). Total cell numbers and leukocyte differentiation were determined by microscopic analysis blinded to the study groups. Cytokines from the BAL fluid supernatant of the first lavage were quantified according to the manufacturer´s instructions using a cytokine multiplex assay (Bioplex®; Bio-Rad Laboratories GmbH, Feldkirchen, Germany).

### Lung Histopathology

Lungs were removed and immersion fixed for 24 h with 4% buffered formaldehyde solution pH 7.0 (Merck KGaA). After embedding in paraffin, tissue sections were cut with a microtome, mounted onto glass slides, and stained.

For histopathological analyses of naïve mice, 3-µm sections were either stained with hematoxylin and eosin (H&E) or immunostained for CD45R/B220 (B cells, monoclonal, 1:1,000, clone RA3-6B2, 553086; BD Biosciences, Heidelberg, Germany) or CD3 (T cells, polyclonal, 1:800; reference A0452; Dako, Santa Clara, CA, USA). Positive immunostaining was visualized using diaminobenzidine, and slides were counterstained with hematoxylin. For analyses of the effects of pulmonary Th2 inflammation, 5-µm tissue sections were either stained with H&E or Masson–Goldner trichrome.

Microscopic analyses were performed (Axiophot; Carl Zeiss Microscopy GmbH, Jena, Germany) in a blinded fashion by a board-certified veterinary pathologist or an anatomist and images were digitized (Color View II camera, CellSens software; Olympus Europa SE Co. KG, Hamburg, Germany).

### Real-Time Quantitative RT-PCR

Gene expression was analyzed as described (31, 57). Lung tissue was homogenized in Trizol (Thermo Fisher Scientific, Dreieich, Germany), and RNA was extracted. Reverse transcription (RT) of total RNA was performed (high-capacity reverse transcription kit; Thermo Fisher Scientific). For quantitative RT-PCR (ABI 7300 instrument; Thermo Fisher Scientific), TaqMan assays (Life Technologies) were applied for the target genes ET_A_, TXA_2_ receptor, and ET-1. TaqMan assay IDs were Mm01243722_m1 (ET_A_), Mm00436917_m1 (TXA_2_ receptor), and Mm00438656_m1 (ET-1). Glyceraldehyde 3-phosphate dehydrogenase (GAPDH) served as internal reference. GAPDH primer sequences were TGTGTCCGTCGTGGATCTGA (forward, 5’ to 3’), CCTGCTTCACCACCTTCTTGA (reverse, 5’ to 3’), and CCGCCTGGAGAAACCTGCCAAGTATG (probe, 5’-FAM to 3’-TAMRA) (57). The relative expression (relative quantity, RQ) of each target gene was quantified using the comparative C_t_ method, with relative expression set to 1 in PBS-treated WT mice (31, 57).

### TXB_2_ and VIP Quantification

Thromboxane B_2_ (TXB_2_) perfusate levels and vasoactive intestinal peptide (VIP) plasma levels were quantified *via* enzyme immunoassay (EIA) according to the respective manufacturer’s guide (TXB_2_ EIA; Cayman Chemical, MI, USA; detection limit 7.8 pg/mL; VIP EIA; Phoenix Europe GmbH, Karlsruhe, Germany; detection limit 0.05 ng/mL).

### Statistical Analysis

For comparison of autoantibody levels, data were analyzed by one-way ANOVA followed by Dunnett’s multiple comparisons test. Dose–response curves were compared using two-way repeated measures ANOVA. Mann–Whitney *U* test was performed for comparison between two groups. *p < 0.05, **p < 0.01, ***p < 0.001.

## Results

### Elevated Anti-ET_B_ Autoantibody Serum Levels in Patients With PAH Secondary to Systemic Sclerosis

**Figure 1:**
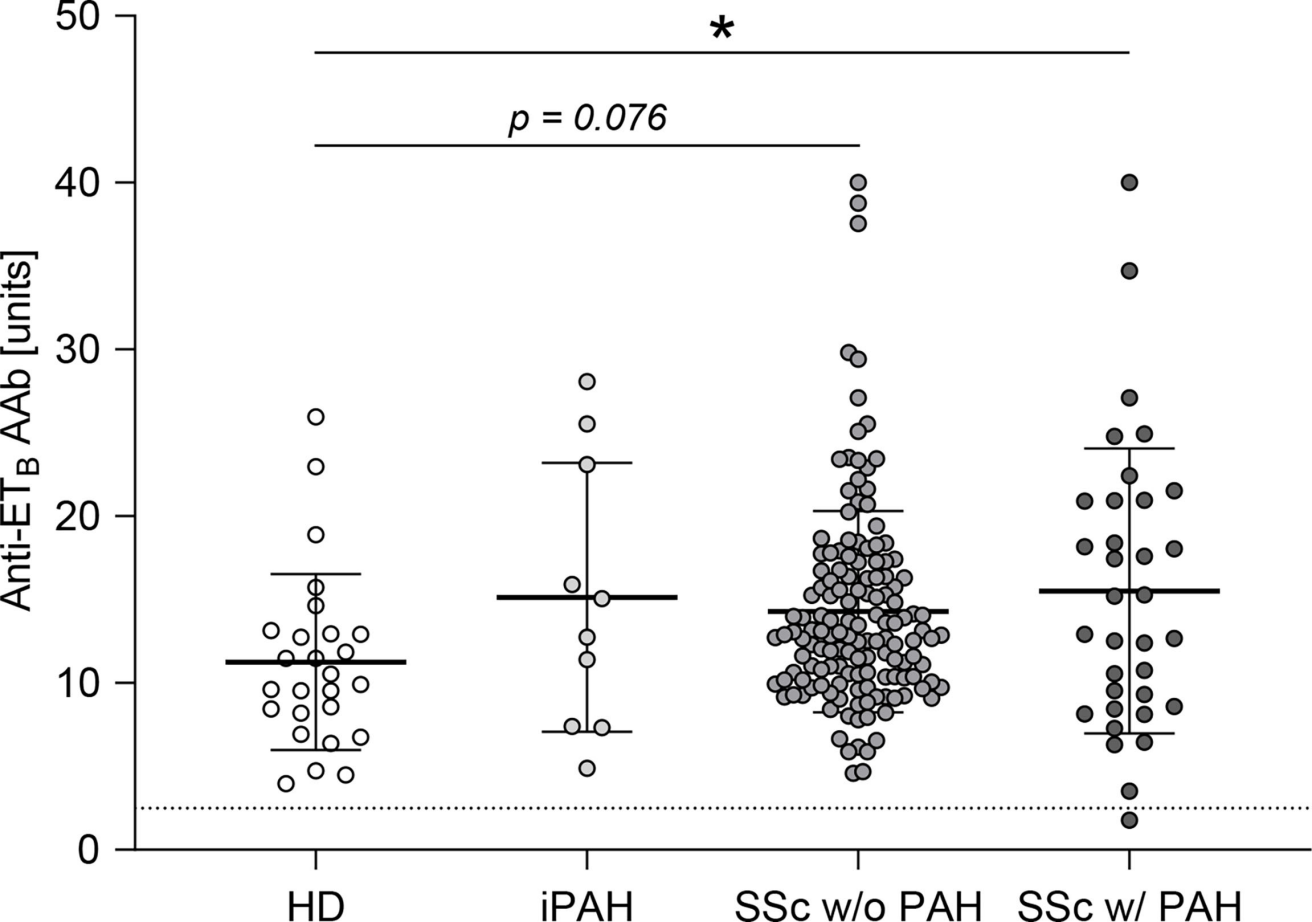
Anti-ET_B_ autoantibody serum levels were elevated in patients with PAH secondary to SSc. Serum levels of anti-endothelin B receptor (ET_B_) autoantibodies (AAb) were quantified in healthy donors (HD), patients with idiopathic pulmonary arterial hypertension (iPAH), and systemic sclerosis (SSc) patients (+/- interstitial lung disease) with or without PAH. Data are expressed as single values with mean ± SD; *N* = 26 (HD) or *N* = 10 (iPAH) or *N* = 143 (SSc w/o PAH) or *N* = 34 (SSc w/PAH). The dotted line indicates the lower detection limit of the ELISA. *p < 0.05 (one-way ANOVA and Dunnett’s multiple comparisons test).

Autoantibodies against ET_A_ were shown to be elevated in SSc-PAH patients (35). Analogously, in this study, anti-ET_B_ autoantibody serum levels were quantified in SSc patients with or without PAH as well as in iPAH patients and healthy donors. Compared to healthy donors, SSc-PAH patients showed increased levels of anti-ET_B_ AAb (**Figure 1**). In SSc patients without PAH as well as in iPAH patients, anti-ET_B_ AAb serum levels were increased by trend when compared to healthy donors (**Figure 1**). However, the relatively small number of iPAH serum samples needs to be considered. Detailed patient characteristics are found in **Supplementary Table 1**.

### Pulmonary Hypertension, Right Ventricular Hypertrophy, and Lymphocytic Alveolitis in _pre_ET^tg^ and ET_B_^-/-^ Mice

To confirm the dual vasomotor role of ET-1 *via* ET_A_ and ET_B_ receptors in the pulmonary vasculature, functional analyses in isolated perfused mouse lungs were performed. In isolated WT lungs, application of the ET_A_ inhibitor BQ-123 resulted in an almost complete reduction of the pulmonary vascular pressure response to ET-1 compared with the solvent control (**Supplementary Figure 1A**), while rescued ET_B_ deficiency resulted in an elevated pulmonary vascular pressure response to ET-1 or the thromboxane receptor agonist U46619 compared to WT controls (**Supplementary Figures 1B, C**).

**Figure 2:**
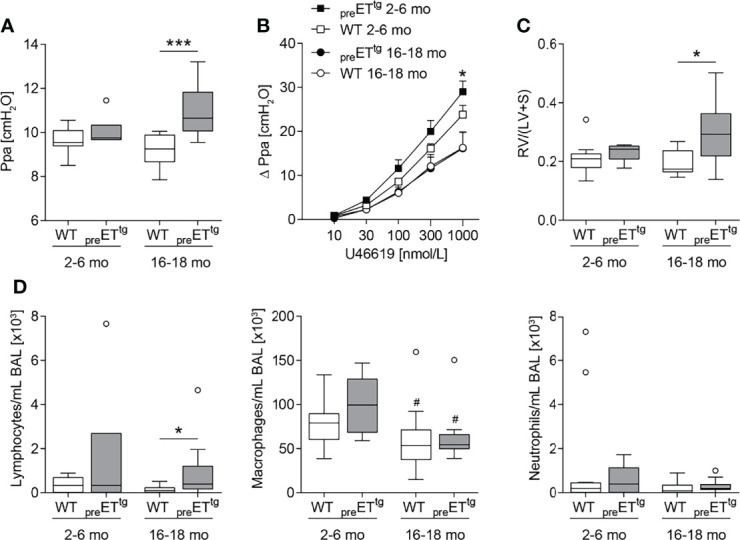
Prepro-endothelin-1 overexpression was age-dependently associated with increased pulmonary arterial pressure, vascular hyperresponsiveness, right ventricular hypertrophy, and increased number of lymphocytes in bronchoalveolar lavage. Lungs and hearts of 2- to 6-month (mo)-old or 16- to 18-mo-old prepro-endothelin-1 overexpressing (_pre_ET^tg^) mice and corresponding wild-type (WT) mice were prepared, or bronchoalveolar lavage (BAL) was performed. **(A)** In isolated perfused and ventilated lungs, under basal conditions, 16- to 18-mo-old _pre_ET^tg^ showed a higher pulmonary arterial pressure (Ppa) compared to WT mice of the same age. **(B)** Pulmonary vascular responsiveness to intravascular application of the thromboxane receptor agonist U46619 was increased in 2- to 6-mo-old _pre_ET^tg^ mice compared to WT controls of the same age. Data (Δ Ppa) represent the difference between the highest pressure response to U46619 and the basal Ppa. **(C)** Fulton index [quotient of right ventricle (RV) and left ventricle (LV) including septum (S)] determined after weighing the cardiac compartments was higher in 16- to 18-mo-old _pre_ET^tg^ compared to WT mice of the same age. **(D)** Analysis of differentially quantified leukocytes in BAL showed increased number of lymphocytes in 16- to 18-mo-old _pre_ET^tg^ compared to WT mice of the same age, whereas macrophages decreased with age, independent of prepro-ET-1 overexpression. In **(A, C, D),** data are represented as *box plots* depicting median, quartiles, and ranges excluding outliers (*open circles*), and analyzed using Mann–Whitney *U* test. ^#^ indicates significant difference between 16- to 18-mo-old vs. 2- to 6-mo-old groups, * indicates significant difference between _pre_ET^tg^ vs. corresponding WT group (as indicated). In **(B)**, values are given as mean and SEM, and analyzed using two-way repeated measures ANOVA, followed by a single Mann–Whitney *U* test between values of _pre_ET^tg^ and WT mice of the same age at the highest dose of U46619 (*). In **(A–C)**, *N* = 5–12; in **(D)**, *N* = 7–17. *^/#^ *p* < 0.05, ***p < 0.001.

To dissect ET-1-specific as well as ET_B_-specific effects on pulmonary inflammation and PAH-associated cardiovascular pathologies independent of additional inflammatory stimuli, we first investigated age-dependent effects in two transgenic mouse models of (i) prepro-ET-1 overexpression (_pre_ET^tg^) and (ii) rescued ET_B_ deficiency (ET_B_^-/-^). In line with the literature (58), young to mature-adult (2–6 months old) _pre_ET^tg^ mice did not show pulmonary hypertension. However, in highly aged (16–18 months old) mice, prepro-ET-1 overexpression resulted in a significant increase in pulmonary arterial pressure compared to corresponding WT controls (**Figure 2A**). Furthermore, 2- to 6-month-old _pre_ET^tg^ mice demonstrated a moderate increase in pulmonary vascular responsiveness to thromboxane receptor agonist U46619 compared to corresponding WT mice, whereas pulmonary vascular responsiveness of 16- to 18-month old mice was comparable within both groups (_pre_ET^tg^ vs. WT) (**Figure 2B**). Cardiac analysis using the Fulton index (right ventricular weight/weight of left ventricle including septum) revealed right ventricular hypertrophy associated with chronic prepro-ET-1 overexpression in 16- to 18-month-old but not in 2- to 6-month-old mice (**Figure 2C**). Long-term overexpression of prepro-ET-1 was also associated with an increase in lymphocyte numbers in the BAL (**Figure 2D**). Independent of prepro-ET-1 overexpression, less BAL macrophages were found in 16- to 18-month-old vs. 2- to 6-month-old mice (**Figure 2D**).

**Figure 3:**
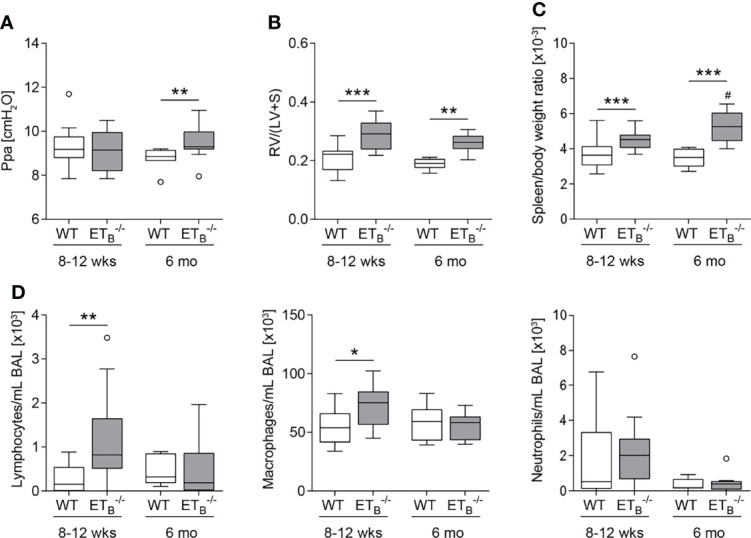
ET_B_ deficiency was age-dependently associated with increased pulmonary arterial pressure, right ventricular hypertrophy, splenomegaly, and increased number of lymphocytes in bronchoalveolar lavage. Lungs, hearts, and spleens of 8- to 12-week (wk)-old and 6-mo-old rescued endothelin B receptor-deficient (ET_B_^-/-^) and corresponding wild-type (WT) mice were removed, or bronchoalveolar lavage (BAL) was performed. **(A)** In isolated perfused and ventilated lungs, under basal conditions, pulmonary arterial pressure (Ppa) was increased in 6-mo-old ET_B_^-/-^ compared to WT mice of the same age. **(B)** Fulton index [ratio of right ventricle (RV) and left ventricle (LV) including septum (S)] determined after weighing the cardiac compartments was higher in ET_B_^-/-^ compared to WT mice. **(C)** Determination of spleen weight related to body weight revealed splenomegaly in ET_B_^-/-^ mice. **(D)** Analysis of differentially quantified leukocytes in BAL revealed increased number of lymphocytes and macrophages in BAL from 8- to 12-wk-old ET_B_^-/-^ vs. WT mice of the same age. Data are represented as *box plots* depicting median, quartiles, and ranges excluding outliers (*open circles*). In **(A–C)**, *N* = 7–28; in **(D)**, *N* = 7–17. ^#^ indicates significant difference in the 6-mo-old vs. the corresponding 8- to 12-wk-old group, * indicates significant difference between ET_B_^-/-^ vs. the corresponding WT group. *^/#^ *p* < 0.05, **p < 0.01, ***p < 0.001 (Mann–Whitney *U* test).

In mature-adult (6 months old) ET_B_^-/-^ mice, basal pulmonary arterial pressure was significantly increased compared to WT mice of the same age (**Figure 3A**). Weight analysis of cardiac compartments revealed that ET_B_^-/-^ mice of both age groups exhibited right ventricular hypertrophy compared with their respective WT counterparts (**Figure 3B**). Furthermore, splenomegaly was present in ET_B_^-/-^ compared to WT mice and was progressive with age (**Figure 3C)** whereas relative liver weight was comparable in all groups (**Supplementary Figure 2A**). Cellular analysis of BAL fluid showed increased numbers of lymphocytes and macrophages in young ET_B_^-/-^ compared to WT mice (**Figure 3D**).

Basal dynamic lung compliance was found to be reduced in ET_B_^-/-^ compared to corresponding WT mice in both age groups (**Supplementary Figure 2B**), in contrast to _pre_ET^tg^ mice, which showed unaltered dynamic lung compliance (**Supplementary Figure 3**).

### Perivascular Lymphoid Infiltrates in ET_B_^-/-^ Lungs

**Figure 4:**
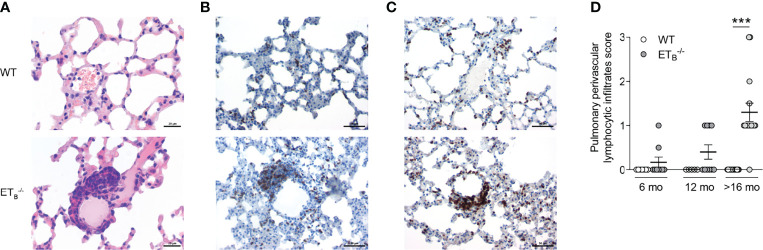
ET_B_ deficiency was associated with peripheral perivascular lymphocytic infiltrates in the lung. Lungs of 6-, 12-, and >16-mo-old rescued endothelin B receptor-deficient (ET_B_^-/-^) and the corresponding wild-type (WT) mice were assessed histologically following hematoxylin and eosin (H&E) stain **(A)** or immunohistochemical stains for CD45R/B220 (B cells; **B**) or CD3 (T cells; **C**). The scale bars represent 20 µm **(A)** or 50 µm **(B, C)**. Representative images of ≥12-mo-old mice are shown; *N* = 20–25 per group **(A)** or *N* = 3–5 per group **(B, C)**. **(D)** H&E-stained lung sections were analyzed and scored (0, no peripheral perivascular infiltrates; 1, mild; 2 moderate; 3, pronounced) by an independent board-certified pathologist, blinded to the study groups. Data are expressed as single values with mean ± SEM; *N* = 7–9 (6-mo-old group), *N* = 5–10 (12-mo-old group), or *N* = 15 (>16-mo-old group). ***p < 0.001 (Mann–Whitney *U* test).

Compared to WT controls, ET_B_^-/-^ mice developed marked perivascular lymphocytic infiltrates (**Supplementary Figure 4**). In ET_B_^-/-^ mice, perivascular lymphocytic infiltrates were particularly observed in the peripheral lung tissue, which were absent from WT lungs (**Figure 4A**). These infiltrates mostly consisted of B cells (**Figure 4B**) and T cells (**Figure 4C**). Both the prevalence and number of these cell clusters increased with age, and infiltrates were present in almost all (14/15) >16 months old ET_B_^-/-^ mice (**Figure 4D**). Such perivascular cell clusters adjacent to small pulmonary arteries were absent in _pre_ET^tg^ mice (**Supplementary Figure 5**) pointing to an immunomodulatory role of ET_B_ in the lungs in the context of pulmonary hypertension.

### Th2 Inflammation Aggravates PAH-Associated Pathologies in ET_B_^-/-^ Lungs

Next, we studied the effects of pulmonary Th2 inflammation as a second inflammatory hit in ET_B_^-/-^ mice. Pulmonary Th2 inflammation was induced *via* systemic ovalbumin sensitization and ovalbumin airway exposure (OVA/OVA).

**Figure 5:**
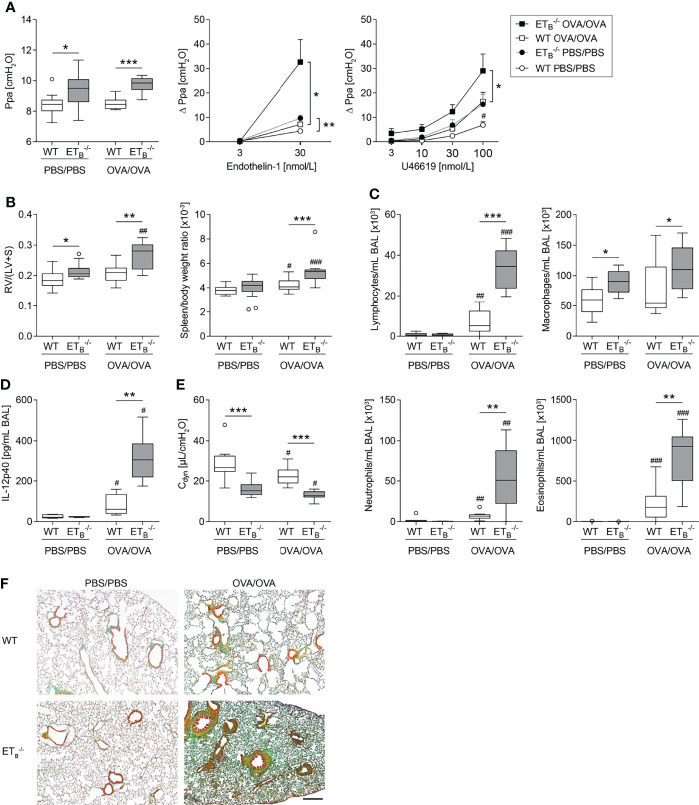
ET_B_ deficiency aggravated Th2-mediated vascular pathologies and inflammation in the lung. Rescued endothelin B receptor-deficient (ET_B_^-/-^) and corresponding wild-type (WT) mice were systemically sensitized with ovalbumin (OVA) (or PBS as control) and repeatedly exposed to aerosolized OVA (OVA/OVA) or PBS (PBS/PBS). Forty-eight hours after the last challenge, lungs, hearts, and spleens of 12-wk-old mice were harvested, or bronchoalveolar lavage (BAL) was performed. **(A)** In isolated perfused and ventilated lungs, pulmonary arterial pressure (Ppa) was measured under basal conditions, and pulmonary vascular responsiveness to increasing concentrations of endothelin-1 or thromboxane receptor agonist U46619 was determined. Data (Δ Ppa) represent the difference between the highest pressure response to the respective stimulus and the basal Ppa. **(B)** Fulton index [quotient of right ventricle (RV) and left ventricle (LV) including septum (S)] was determined after weighing the cardiac compartments (left) and spleen weight was determined and related to body weight (right). **(C)** Leukocytes were differentially quantified in BAL. **(D)** IL-12p40 was determined in BAL (lower detection limit was 0.54 pg/mL). **(E)** In isolated perfused and ventilated mouse lungs, dynamic lung compliance (C_dyn_) was measured. **(F)** Lung tissue sections were stained with Masson–Goldner trichrome that revealed more pronounced pulmonary collagen deposition in ET_B_^-/-^ than WT mice after OVA/OVA treatment. The scale bar represents 100 µm and is valid for all photomicrographs. Representative images (*N* = 7 per group) are shown. In **(A left, B–E),** data are represented as *box plots* depicting median, quartiles, and ranges excluding outliers (*open circles*), and analyzed using Mann–Whitney *U* test. ^#^ indicates significant difference between OVA/OVA vs. the corresponding PBS/PBS group, * indicates significant difference between ET_B_^-/-^ vs. the corresponding WT group. In **(A middle-right)**, values are given as mean and SEM, and analyzed using two-way repeated measures ANOVA (*). In **(A right)**, additional Mann–Whitney *U* test was performed comparing values of ET_B_^-/-^ and WT mice treated with PBS at the highest dose of U46619 (#). *N* = 5–14 **(A–C, E)** or *N* = 3–6 **(D)**. *^/#^ *p* < 0.05, **^/##^ *p* < 0.01, ***^/###^ *p* < 0.001.

Pulmonary arterial pressure was elevated in ET_B_^-/-^ compared to WT mice as described before, independent of pulmonary Th2 inflammation (**Figure 5A**). Following OVA/OVA treatment, pulmonary vascular hyperresponsiveness to ET-1 and U46619 was highly increased in ET_B_^-/-^ mice compared to WT mice (**Figure 5A**). Pulmonary vascular hyperresponsiveness to serotonin, however, was not increased in ET_B_^-/-^ as compared to WT mice (**Supplementary Figure 6A**).

Importantly, pulmonary Th2 inflammation aggravated right ventricular hypertrophy as well as splenomegaly in ET_B_^-/-^ mice compared to WT controls (**Figure 5B**). Liver weight in relation to body weight was comparable in all groups (**Supplementary Figure 6B**).

The Th2-mediated inflammatory cell influx into the lung was increased in ET_B_^-/-^ mice as reflected by elevated BAL cell numbers including lymphocytes, neutrophils, and eosinophils (**Figure 5C**) and as revealed by more pronounced perivascular leukocyte infiltrates in ET_B_^-/-^ lungs (**Supplementary Figure 7**). While BAL Th2 cytokines IL-4, IL-5, and IL-13 in OVA/OVA-treated ET_B_^-/-^ mice were comparable with the respective WT mice (**Supplementary Table 2**), IL-12 subunit p40 (IL-12p40) levels were greatly increased in BAL of ET_B_^-/-^ mice (**Figure 5D**).

### ET_B_ Deficiency Aggravates Th2-Mediated Collagen Deposition in the Lung

Th2 immune responses have been associated with pulmonary collagen deposition (59) and IL-12p40 is believed to possess profibrotic properties in the lung (60). Dynamic lung compliance was lowest in ET_B_^-/-^ mice after induction of pulmonary Th2 inflammation (**Figure 5E**). Accordingly, histological analyses of Masson–Goldner trichrome-stained lung slices revealed more pronounced collagen deposition in ET_B_^-/-^ lungs than in WT lungs following OVA/OVA treatment (**Figure 5F**).

### ET_B_ Mediates Thromboxane Release Evoked by ET-1

**Figure 6:**
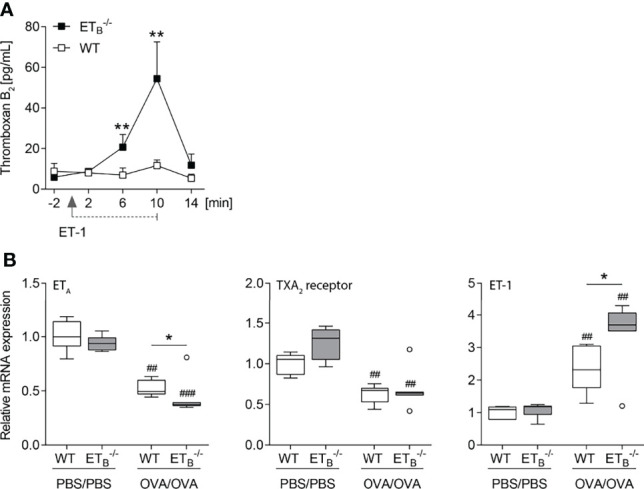
ET-1**-**mediated thromboxane release is increased in ET_B_^-/-^ mice. **(A)** In isolated perfused lungs of 10- to 12-wk-old rescued endothelin B receptor-deficient (ET_B_^-/-^) and corresponding wild-type (WT) mice, perfusate samples were collected 2 min before application of 100 nmol/L ET-1 (for 10 min), and 2, 6, 10, and 14 min after the start of ET-1 application, and TXB_2_ levels were determined. The detection limit was 7.8 pg/ml. Values are given as mean and SEM (*N* = 7), and analyzed using Mann–Whitney *U* tests at each time point comparing both groups. **(B)** ET_B_^-/-^ and corresponding WT mice were systemically sensitized with OVA (or PBS as control) and repeatedly exposed to aerosolized OVA (OVA/OVA) or PBS (PBS/PBS). Forty-eight hours after the last inhalative OVA challenge, lungs of 15- to 17-wk-old mice were isolated for mRNA expression analyses by quantitative PCR. Relative quantification of mRNA was performed using the comparative C_t_ method. Data are represented as *box plots* depicting median, quartiles, and ranges excluding outliers (*open circles*), and analyzed using Mann–Whitney *U* test; *N* = 6**–**7 per group. ^#^ indicates significant difference between OVA/OVA vs. the corresponding PBS/PBS group, * indicates significant difference between ET_B_^-/-^ vs. the corresponding WT group. *p < 0.05, **^/##^ *p* < 0.01, ^###^ *p* < 0.001.

To mechanistically dissect the pronounced increase in pulmonary vascular responsiveness to ET-1 in ET_B_^-/-^ mice (**Figure 5A**), we indirectly assessed pulmonary vascular TXA_2_ release in isolated mouse lungs *via* quantification of stable TXB_2_ in the perfusate before and after intravascular application of ET-1. Indeed, vascular thromboxane release following ET-1 application was highly elevated in ET_B_^-/-^ lungs (**Figure 6A**).

Increased vascular hyperresponsiveness secondary to ET_A_ and/or TXA_2_ receptor upregulation, however, was ruled out *via* mRNA expression analyses. In ET_B_^-/-^ lungs, ET_A_ mRNA expression was downregulated while TXA_2_ receptor mRNA expression was comparable (**Figure 6B**). Of note, rescued ET_B_ deficiency led to increased pulmonary ET-1 expression following induction of pulmonary Th2 inflammation (**Figure 6B**).

To investigate a potential pathomechanistic link between the endothelin system in pulmonary Th2 inflammation and the VIP, VIP plasma levels were quantified. Neither rescued ET_B_ deficiency nor OVA/OVA treatment had an effect on VIP plasma levels (**Supplementary Figure 8**).

## Discussion

In our study, we evaluated anti-ET_B_ AAb in PAH patients for the first time and found increased levels in SSc-PAH patients. Furthermore, we characterized the immunomodulatory role of ET_B_ in the context of PAH using a mouse model of PAH due to rescued ET_B_ deficiency. Our data point to an important role of ET_B_ in immune homeostasis, with functional ET_B_ deficiency unleashing PAH development under inflammatory conditions.

ET_B_ deficiency is associated with defective ET-1 clearance (27, 41–43), and increased levels of plasma ET-1 (44–46). In order to distinguish ET-1- and ET_B_-dependent effects in ET_B_^-/-^ mice, we studied a second transgenic mouse model in parallel, namely, prepro-endothelin-1 overexpressing (_pre_ET^tg^) mice.

Here, we show that pulmonary hypertension, pulmonary vascular hyperresponsiveness, and right ventricular hypertrophy were present in _pre_ET^tg^ as well as in ET_B_^-/-^ mice, arguing for ET-1-specific effects. In _pre_ET^tg^ mice, both pulmonary hypertension and right ventricular hypertrophy, however, were exclusively detected in highly aged (≥16 months old) mice, possibly as a result of decreasing NO-mediated compensatory effects with increasing age (61). In contrast, in ET_B_^-/-^ mice, PAH-associated alterations were generally observed at a younger age than in _pre_ET^tg^ mice, which may be the result of synergistic unfavorable effects of ET_B_ deficiency and consecutive defective clearance of ET-1. Yet, as opposed to the characteristic findings in PAH patients, relevant pulmonary arterial remodeling was absent in both transgenic mouse lines, suggesting that the observed pulmonary hypertensive phenotypes are primarily driven by an increased vascular tone due to an imbalance of vasoconstrictive and vasodilatory mechanisms, rather than by extensive vascular remodeling in the pulmonary circulation.

Increased numbers of lymphocytes were found in BAL of both transgenic mouse models, again pointing to ET-1 as causative trigger. This finding is in line with previously described chronic lymphocytic lung inflammation as a result of prepro-ET-1 overexpression (58).

Of note, pronounced peripheral perivascular cluster of lymphoid infiltrates were exclusively found in ET_B_^-/-^ lungs, arguing for a dysregulation of the immune system due to the loss of ET_B_. The perivascular space is a unique lung compartment, which might have been underestimated (62). Capillaries as well as lymphatic vessels from the periphery are found in the perivascular space, predominantly around the pulmonary arteries. This compartment is rather inactive in healthy lungs, but gains major significance in many types of lung inflammation. It is hypothesized that in certain conditions, inflammatory mediators induce extravasation of fluid and leukocytes from the periarterial capillaries, leading to thick cellular cuffs around the pulmonary arteries (63, 64). This defense mechanism may contribute to secondary lesions or processes such as the development of tertiary lymphoid tissue.

The data presented here give a strong hint that ET_B_ is involved in the regulation of perivascular infiltration of pulmonary arteries. This finding is of specific interest as perivascular infiltrates and lymphoid tissue are commonly found in lungs of PAH patients and in preclinical models of pulmonary hypertension (2, 9–12).

As previously shown by us and others, induction of pulmonary Th2 inflammation in mice induces relevant PAH-associated features such as perivascular inflammation, hyperresponsiveness of the pulmonary vasculature to vasoconstrictive stimuli, complex pulmonary arterial remodeling, and increase in right ventricular systolic pressure (2, 16–23). Importantly, a key role of Th2 inflammation in the pathogenesis of pulmonary hypertension has been demonstrated in lung-specific IL-13-overexpressing mice, which develop spontaneous pulmonary hypertension, pulmonary arterial remodeling, and right ventricular hypertrophy (22). Interestingly, also in Fra-2 transgenic mice, a well-described model of SSc-PAH and interstitial lung disease, a strong underlying Th2 phenotype is present (56, 65).

Here, we analyzed the effects of pulmonary Th2 inflammation as a second hit in ET_B_^-/-^ mice. Notably, both pulmonary vascular hyperresponsiveness and right ventricular hypertrophy were aggravated secondary to Th2 inflammation in ET_B_^-/-^ mice. Moreover, in ET_B_^-/-^ lungs, pulmonary perivascular inflammation and collagen deposition were increased.

On the cytokine level, IL-12 subunit p40 (IL-12p40) levels were largely increased in BAL of ET_B_^-/-^ mice, whereas Th2 cytokines were similar in ET_B_^-/-^ and WT mice. Increased IL-12p40 levels are notable with respect to the exaggerated PAH phenotype in ET_B_^-/-^ mice as well as the increased pulmonary collagen deposition, since PAH patients show elevated levels of circulating IL-12p40 (12). Analogously, IL-12p40 serum levels are increased in mice deficient for chemokine receptor CCR7, which develop pulmonary hypertension, pulmonary arterial remodeling, and perivascular lymphoid infiltrates in the lung (12). Moreover, IL-12p40 has been identified as a central profibrotic mediator in murine lung fibrosis (60).

Pulmonary vascular hyperresponsiveness to the stimuli ET-1 and TXA_2_ analog U46619 was shown to be aggravated in ET_B_^-/-^ lungs compared to the WT lungs. Interestingly, pulmonary vascular hyperresponsiveness to serotonin was unchanged in ET_B_^-/-^ lungs, arguing for stimulus-specific alterations of the here assessed vasomotor responses. Mechanistically, ET-1-evoked hyperresponsiveness was most likely based on increased TXA_2_ release following vascular ET-1 application in ET_B_^-/-^ mice, as indicated here by the elevated TXB_2_ perfusate levels.

Increased responsiveness as a result of ET_A_ and/or TXA_2_ receptor upregulation, however, was ruled out in this study. In fact, ET_A_ was downregulated in ET_B_^-/-^ lungs following induction of Th2 inflammation, possibly as a counter-response to the increase in local ET-1 expression in ET_B_^-/-^ lungs. Taken together, both the upregulation of ET-1 expression and the increased release of IL-12p40 may have contributed to the here observed exaggeration of the PAH phenotype in ET_B_^-/-^ mice following induction of pulmonary Th2 inflammation.

It can be assumed that the anti-inflammatory effects of ET_B_ signaling described in this study are primarily mediated *via* ET_B_ receptor activation on vascular and inflammatory cells. In contrast, secondary immunomodulatory effects in response to ET_B_-regulated sodium and water reabsorption in the kidney are unlikely to underlie the detected anti-inflammatory properties of ET_B_ in the lung. Specifically, ET_B_ exerts natriuretic functions (66), and collecting duct-specific deficiency of ET_B_ accordingly causes systemic hypertension with decreased urinary aldosterone excretion and plasma renin activity (67). Reduced activation of the renin–angiotensin–aldosterone system is, however, characteristically associated with mitigated inflammation (68). The same holds true for natriuretic peptides, which are abundantly released upon volume expansion (69) and have protective immunomodulatory properties (70). These anti-inflammatory effects of renal ET_B_ deficiency stand in contrast to the pro-inflammatory effects in the lung detected in our study. Therefore, the anti-inflammatory role of ET_B_ in the lung seems unrelated to its natriuretic function.

Our finding that Th2 inflammation as a second hit augments hallmarks of PAH is in line with previous findings in mice expressing a hypomorphic bone morphogenetic protein receptor type 2 (BMPR2) transgene, which showed an increase in right ventricular systolic pressure following induction of a Th2 immune response (23). As Th2-mediated aggravation of PAH phenotypes has been repeatedly shown, it is tempting to speculate that mediators of Th2 signaling may serve as potential targets in PAH. This needs to be evaluated in further studies.

PAH may occur in SSc patients with or without accompanying interstitial lung disease and/or digital ulcers (71, 72). The endothelin system is believed to play a central role in SSc-PAH, and SSc-PAH patients benefit from ERA treatment (24, 71, 72). ERAs are further indicated to treat SSc-related digital ulcers (72). Additional SSc-associated complications seem to involve the endothelin system. Alveolitis is frequently observed in SSc patients (73, 74). Intriguingly, alveolitis was also experimentally induced in mice treated with anti-ET_A_ AAb and anti-angiotensin II type 1 receptor (AT_1_R) AAb-positive IgG derived from SSc patients (75). Moreover, expression of type I collagen in fibroblasts following treatment with IgG from SSc patients correlated with anti-ET_A_ AAb levels, suggesting an underlying role of the endothelin system in SSc-associated fibrosis (75), as also discussed elsewhere (76).

The immunomodulatory actions of ET_B_ shown here may be of relevance for the early phase of PAH, in which inflammation, endothelial dysfunction, and hyperresponsiveness of the pulmonary vasculature are believed to play relevant mechanistic roles. In the chronic disease state, reflected by profound remodeling of the pulmonary arteries, ET_B_, however, may play a less prominent role, as indicated by the fact that ET_A_-selective blockers do not appear to be superior to dual ET_A_/ET_B_ therapy in established PAH (77, 78). Prospective clinical trials comparing selective ERA (inhibition of ET_A_) head-to-head against dual ERA (combined inhibition of ET_A_/ET_B_) therapy at early disease time points may be required to identify a potential advantage of selective ERA therapy in PAH.

In conclusion, our data show an anti-inflammatory role of ET_B_. ET_B_ deficiency as a single hit is associated with spontaneous formation of marked lymphoid infiltrates in the perivascular space of the lung, in addition to pulmonary hypertension, pulmonary vascular hyperresponsiveness, and right ventricular hypertrophy. Th2 inflammation as a second hit aggravates PAH-associated pathologies in ET_B_^-/-^ mice. The pathogenic role of anti-ET_B_ AAb in SSc-PAH needs to be evaluated in further studies.

## Data Availability Statement

The raw data supporting the conclusions of this article will be made available by the authors, without undue reservation.

## Ethics Statement

The studies involving human participants were reviewed and approved by the ethics committee (Charité - Universitätsmedizin Berlin, Germany; EA1/179/17). The patients/participants provided their written informed consent to participate in this study. The animal study was reviewed and approved by institutional authorities of the Charité – Universitätsmedizin Berlin, Germany, and the Local State Office of Health and Social Affairs Berlin (LAGeSo; Berlin, Germany).

## Author Contributions

CT and MW conceived and designed the research. CT, CG, TT, OK, BG, JN, and JHe performed experiments. CT, CG, JL, JHö, TT, OK, BG, JN, BO, AG, HH, ES, and MW analyzed data. All authors interpreted the results of the experiments. CT, CG, JL, JHö, TT, OK, and ES prepared figures. CT and JL drafted the manuscript. All authors edited and revised the manuscript and approved the final version of the manuscript.

## Funding

This study received funding from the German Research Foundation (SFB-TR84, C6/C9 to MW and Z01b to AG) and Actelion Pharmaceuticals Germany GmbH. The funders were not involved in the study design, collection, analysis, interpretation of data, the writing of this article or the decision to submit it for publication.

## Conflict of Interest

CT received funding for research from Deutsche Gesellschaft für Pneumologie, Bayer HealthCare, Boehringer Ingelheim, and for lectures from Actelion Pharmaceuticals, Boehringer Ingelheim. HH is CEO of CellTrend GmbH, Luckenwalde, Germany. MW received funding for research from Deutsche Forschungsgemeinschaft, Bundesministerium für Bildung und Forschung, Deutsche Gesellschaft für Pneumologie, European Respiratory Society, Marie Curie Foundation, Else Kröner Fresenius Stiftung, CAPNETZ STIFTUNG, International Max Planck Research School, Actelion, Bayer Health Care, Biotest AG, Boehringer Ingelheim, NOXXON Pharma, Pantherna, Quark Pharma, Silence Therapeutics, Vaxxilon, and for lectures and advisory from Actelion, Alexion, Aptarion, Astra Zeneca, Bayer Health Care, Berlin Chemie, Biotest, Boehringer Ingelheim, Chiesi, Glaxo Smith Kline, Insmed, Novartis, Teva and Vaxxilon.

The remaining authors declare that the research was conducted in the absence of any commercial or financial relationships that could be construed as a potential conflict of interest.

## Publisher’s Note

All claims expressed in this article are solely those of the authors and do not necessarily represent those of their affiliated organizations, or those of the publisher, the editors and the reviewers. Any product that may be evaluated in this article, or claim that may be made by its manufacturer, is not guaranteed or endorsed by the publisher.

## Abbreviations

AT_1_R, angiotensin II type 1 receptor; AAb, autoantibody; BAL, bronchoalveolar lavage; BMPR2, bone morphogenetic protein receptor type 2; C_dyn_, dynamic lung compliance; ET-1, endothelin-1; ET_A_, endothelin A receptor; ET_B_, endothelin B receptor; ERA, endothelin receptor antagonist; H&E, hematoxylin and eosin; IgG, immunoglobulin G; IL, interleukin; IL-12p40, IL-12 subunit p40; NO, nitric oxide; OVA, ovalbumin; OVA/OVA, OVA-sensitized and OVA-challenged; PAH, pulmonary arterial hypertension; PBS, phosphate buffered saline; PBS/PBS, non-sensitized and non-challenged (PBS-treated); PASMCs, pulmonary arterial smooth muscle cells; Ppa, pulmonary arterial pressure; SSc, systemic sclerosis; Th2, T-helper type 2; TNF-α, tumor necrosis factor alpha; TXA_2_, thromboxane A_2_; TXB_2_, thromboxane B_2_; VIP, vasoactive intestinal peptide.
