# Supplementary material for: Endothelin B Receptor Immunodynamics in Pulmonary Arterial Hypertension
Source: Front Immunol. 2022 Jun 9;13:895501. doi: 10.3389/fimmu.2022.895501 (PMC9221837; doi:10.3389/fimmu.2022.895501)
Supplement: Supplementary file 1 [file DataSheet_1.pdf]

Supplementary Material

Supplementary Figures and Tables

Supplementary Figure 1

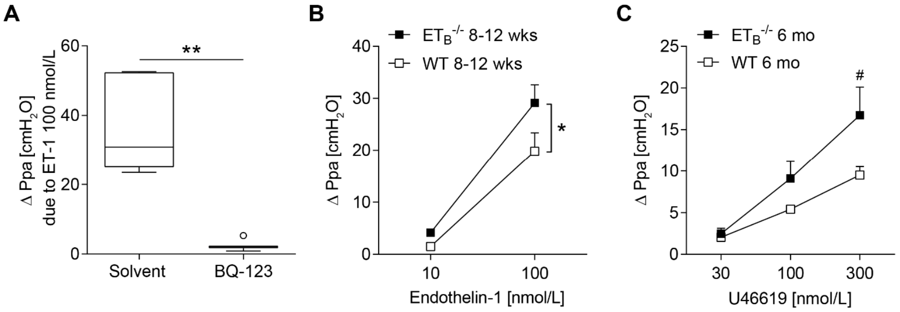

Supplementary Figure 1: ET_A inhibition reduced and ET_B deficiency increased pulmonary vascular responsiveness. Lungs of 8-12 weeks (wks) and 6 months (mo) old rescued endothelin B receptor-deficient ($ET_B^{-/-}$) and corresponding wild-type (WT) mice were prepared. **(A)** In isolated perfused and ventilated lungs of 8-12 wks old WT mice, application of the endothelin A receptor inhibitor BQ-123 (8 µM) almost completely reduced the pulmonary arterial pressure response to endothelin-1 (ET-1). **(B-C)** In isolated perfused lungs of 8-12 wks or 6 mo old mice, pulmonary vascular responsiveness to ET-1 **(B)** or thromboxane analog U46619 **(C)** was increased in $ET_B^{-/-}$ compared to WT mice. Data (Δ Ppa) represent the difference between the highest pressure response to the respective stimulus and the basal Ppa. In **(A)**, data are represented as *box plots* depicting median, quartiles, and ranges excluding outliers (*open circles*), and analyzed using Mann-Whitney U test; N=5-7. In **(B-C)**, values are given as mean and SEM, and analyzed using two-way repeated measures ANOVA (*); N=7 each group. In **(C)**, additional Mann-Whitney U test was performed comparing values of both groups at the highest dose of U46619 (#). $^{*/\#}p<0.05$, $^{**}p<0.01$.

Supplementary Figure 2

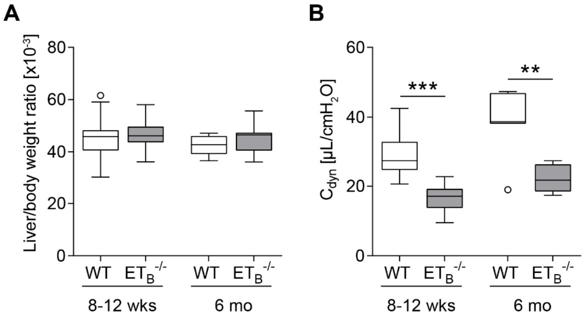

Supplementary Figure 2: ET_B deficiency was associated with reduced dynamic lung compliance. Lungs of 8-12 weeks (wks) and 6 months (mo) old rescued endothelin B receptor-deficient ($ET_B^{-/-}$) and corresponding wild-type (WT) mice were prepared and livers were removed. **(A)** Liver weight related to body weight was similar in all groups. **(B)** In isolated perfused and ventilated lungs, dynamic lung compliance (C_{dyn}) was decreased in $ET_B^{-/-}$ compared to WT mice, independent of age. Data are represented as *box plots* depicting median, quartiles, and ranges excluding outliers (*open circles*); N=7-28. **p<0.01, ***p<0.001 (Mann-Whitney U test).

Supplementary Figure 3

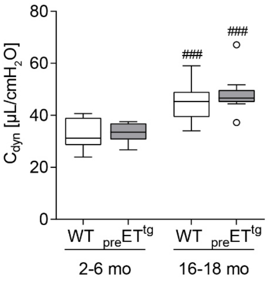

Supplementary Figure 3: Dynamic lung compliance in prepro-endothelin-1 overexpressing mice. Lungs of 2-6 months (mo) or 16-18 mo old prepro-endothelin-1 overexpressing ($_{pre}ET^{tg}$) mice and corresponding wild-type (WT) mice were prepared. In isolated perfused and ventilated lungs, dynamic lung compliance was comparable in both groups of the same age. Data are represented as *box plots* depicting median, quartiles, and ranges excluding outliers (*open circles*); N=7-12; $^{\#}$indicates significant difference between 16-18 mo vs. 2-6 mo old groups. $^{\#\#\#}p<0.001$ (Mann-Whitney U test).

Supplementary Figure 4

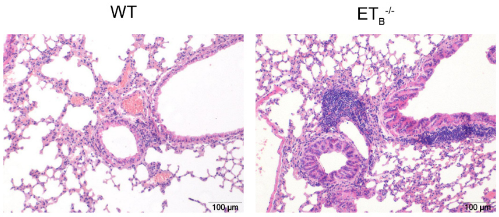

Supplementary Figure 4: ET_B deficiency was associated with perivascular lymphocytic infiltrates in the lung. Lungs of rescued endothelin B receptor-deficient ($ET_B^{-/-}$) and corresponding wild-type (WT) mice were harvested and lung tissue sections stained with hematoxylin and eosin. Representative images (N=20-25 per group) of ≥12 mo old mice are shown (identical lungs as shown in Figure 4, here with lower magnification for a broader overview). Scale bars represent 100 µm.

Supplementary Figure 5

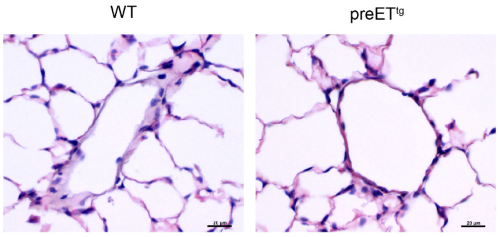

Supplementary Figure 5: The perivascular space of small pulmonary arteries was unaltered in prepro-endothelin-1 overexpressing mice compared to wild-type mice. Lungs of prepro-endothelin-1 overexpressing ($_{pre}ET^{tg}$) mice and corresponding wild-type (WT) mice were harvested and lung tissue sections stained with hematoxylin and eosin. Representative images (N=23-32 per group) of ≥12 mo old mice are shown. Scale bars represent 20 µm.

Supplementary Figure 6

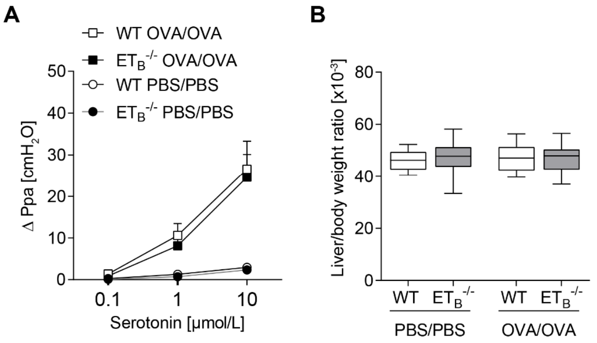

Supplementary Figure 6: ET$_B$ deficiency did not affect Th2-mediated pulmonary vascular hyperresponsiveness to serotonin. Rescued endothelin B receptor-deficient (ET$_B$$^{-/-}$) and corresponding wild-type (WT) mice were systemically sensitized with OVA (or PBS as control) and repeatedly exposed to aerosolized OVA (OVA/OVA) or PBS (PBS/PBS). Forty-eight hours after the last challenge, lungs were prepared and livers were removed. **(A)** In isolated perfused and ventilated lungs, pulmonary vascular responsiveness to increasing concentrations of serotonin was determined. Data (Δ Ppa) represent the difference between the highest pressure response to serotonin and the basal Ppa. **(B)** Liver weight was determined and related to body weight. In **(A)**, values are given as mean and SEM, and analyzed using two-way repeated measures ANOVA (N=5-7). In **(B)**, data are represented as *box plots* depicting median, quartiles, and ranges excluding outliers (*open circles*), and analyzed using Mann-Whitney U test (N=13-14).

Supplementary Figure 7

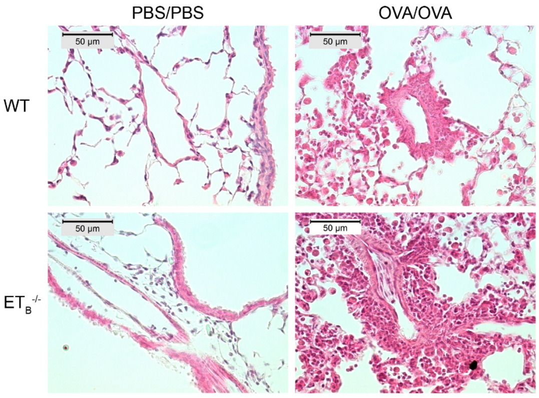

Supplementary Figure 7: ET$_B$ deficiency aggravated Th2-induced perivascular inflammation in the lung. Rescued endothelin B receptor-deficient (ET$_B^{-/-}$) and corresponding wild-type (WT) mice were systemically sensitized with OVA (or PBS as control) and repeatedly exposed to aerosolized OVA (OVA/OVA) or PBS (PBS/PBS). Forty-eight hours after the last challenge, lungs were removed and fixed. Lung tissue sections were stained with hematoxylin and eosin. Representative images (n=7) per group are shown.

Supplementary Figure 8

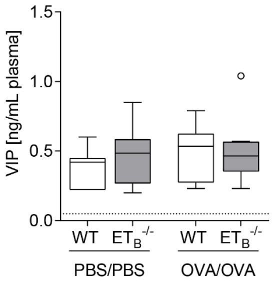

Supplementary Figure 8: Quantification of plasma levels of vasoactive intestinal peptide (VIP). Rescued endothelin B receptor-deficient ($ET_B^{-/-}$) and corresponding wild-type (WT) mice were systemically sensitized with OVA (or PBS as control) and repeatedly exposed to aerosolized OVA (OVA/OVA) or PBS (PBS/PBS). Forty-eight hours after the last challenge, plasma samples were collected and stored at -80°C. VIP levels in plasma were quantified by EIA. The detection limit was 0.05 ng/mL (dotted line). Data are represented as *box plots* depicting median, quartiles, and ranges excluding outliers (*open circles*), and analyzed using Mann-Whitney U test (N=7-8 per group).

Supplementary Table 1

Clinical characteristics

Characteristic	HD	iPAH	SSc w/o PAH +/- ILD	SSc w/ PAH +/- ILD
Patients (no.)	26	10	143	34
Median age (range) – years	30 (23 – 82)	65 (44 – 77)	59 (26 – 87)	69 (41 – 91)
Female sex – no. (%)	15 (58)	8 (80)	115 (80)	28 (82)
Laboratory findings				
Anti-nuclear antibody (ANA) – %		10	92	91
Anti-Scl70 antibody (anti-topoisomerase 1-antibody) – %		0	39	26
Anti-centromere antibody – %		0	32	55
Anti-RNA polymerase III antibody – %		0	7	9
Median NT-proBNP (range) – ng/L		345 (200 – 22764)	113 (10 – 7968)	956 (113 – 7510)
SSc disease course				
Median disease duration (range) – years			7 (0 – 39)	7 (0 – 54)
Raynaud symptoms – %			85	97
Limited cutaneous Systemic Sclerosis (lcSSc) – %			62	68
Diffuse cutaneous Systemic Sclerosis (dcSSc) – %			32	32
Systemic Sclerosis sine scleroderma – %			6	0
Interstitial lung disease (ILD) – %			48	56
Scleroderma renal crisis (SRC) – %			4	6
Digital ulcers – %			42	26
Cardiopulmonary parameters				
Median LVEF (range) – %		63 (55 – 75)	60 (22 – 91)	60 (50 – 68)
Median $DLCO_{SB}$ (range) – %		63 (21 – 93)	56 (17 – 95)	36 (19 – 77)
Median FEV_1 (range) – %		72 (50 – 106)	91 (30 – 133)	80 (33 – 133)
Median FVC (range) – %		77 (50 – 107)	94 (29 – 138)	82 (47 – 150)
Therapy				
Calcium channel blockers – %		10	43	15
Median no. systemic PAH-specific therapy (range)		2 (0 – 3)		1 (0 – 3)
PAH-specific monotherapy – %		20		53
PAH-specific dual therapy – %		50		35
PAH-specific tripple therapy – %		20		3
Intermitting prostacyclin analoga infusion – %		20	50	18

Disease duration refers to the time since first non-Raynaud symptom. Abbreviations: $DLCO_{SB}$, Single-breath diffusing capacity for carbon monoxide; FEV_1, forced expiratory volume per second; FVC, forced vital capacity; HD, healthy donors; ILD, interstitial lung disease; iPAH, idiopathic pulmonary arterial hypertension; L, liter; LVEF, left ventricular ejection fraction; no., number; NT-proBNP, N-terminal-pro-brain natriuretic peptide; PAH, pulmonary arterial hypertension; SSc, Systemic Sclerosis; w/, with; w/o, without.

Supplementary Table 2

Cytokine levels in bronchoalveolar lavage

Cytokine	Detection range	WT PBS/PBS	$ET_B^{-/-}$ PBS/PBS	WT OVA/OVA	$ET_B^{-/-}$ OVA/OVA
IL-1β	*6.24-23,002 pg/mL*	6.24 ± 0	6.24 ± 0	7.64 ± 0.90	7.02 ± 0.50
IL-2	*0.91.3,939 pg/mL*	1.90 ± 0.13	1.62 ± 0.71	2.08 ± 0.31	1.36 ± 0.13 *
IL-4	*1.01-14,576 pg/mL*	1.01 ± 0	1.01 ± 0	7.01 ± 4.28 #	4.15 ± 1.78
IL-5	*0.45-1,804 pg/mL*	0.51 ± 0.06	0.57 ± 0.09	7.43 ± 2.20 ##	4.31 ± 1.15 #
IL-13	*3.62 – 58,768 pg/mL*	4.56 ± 0.62	4.15 ± 0.53	25.04 ± 7.79 #	21.78 ± 5.50 #
KC	*2.4 – 41,653 pg/mL*	1,261 ± 481	953 ± 480	812 ± 282	780 ± 114
RANTES	*5.27 – 16,749 pg/mL*	10.25 ± 2.04	9.33 ± 2.99	9.09 ± 1.50	9.36 ± 0.74
G-CSF	*0.73 – 3,375 pg/mL*	29.60 ± 8.34	19.77 ± 4.77	92.70 ± 29.00	142 ± 25.84 #

Data are mean values ± SEM (pg/mL); N=3-5 (PBS/PBS) or N=6 (OVA/OVA). # indicates significant difference between OVA/OVA vs. corresponding PBS/PBS group, * indicates significant difference between $ETB^{-/-}$ vs. corresponding WT group. */#p<0.05, ##p<0.01 (Mann-Whitney U test). Abbreviations: G-CSF, granulocyte-colony-stimulating factor; IL, interleukin; KC, keratinocyte chemoattractant; RANTES, regulated upon activation normal T cell expressed and secreted.
